# High-Resolution Computed Tomography and Pulmonary Function Findings of Occupational Arsenic Exposure in Workers

**DOI:** 10.4274/balkanmedj.2016.0795

**Published:** 2017-05-15

**Authors:** Recai Ergün, Ender Evcik, Dilek Ergün, Begüm Ergan, Esin Özkan, Özge Gündüz

**Affiliations:** 1 Clinic of Chest Diseases, Dışkapı Yıldırım Beyazıt Training and Research Hospital, Ankara, Turkey; 2 Clinic of Radiology, Ankara Occupational Diseases Hospital, Ankara, Turkey; 3 Clinic of Chest Diseases, Ankara Occupational Diseases Hospital, Ankara, Turkey; 4 Department of Chest Diseases, Dokuz Eylül University School of Medicine, İzmir, Turkey; 5 Clinic of Biochemistry, Ankara Occupational Diseases Hospital, Ankara, Turkey; 6 Department of Dermatology, Ufuk University School of Medicine, Ankara, Turkey

**Keywords:** arsenic, occupational disease, pulmonary function

## Abstract

**Background:**

The number of studies where non-malignant pulmonary diseases are evaluated after occupational arsenic exposure is very few.

**Aims:**

To investigate the effects of occupational arsenic exposure on the lung by high-resolution computed tomography and pulmonary function tests.

**Study Design:**

Retrospective cross-sectional study.

**Methods:**

In this study, 256 workers with suspected respiratory occupational arsenic exposure were included, with an average age of 32.9±7.8 years and an average of 3.5±2.7 working years. Hair and urinary arsenic levels were analysed. High-resolution computed tomography and pulmonary function tests were done.

**Results:**

In workers with occupational arsenic exposure, high-resolution computed tomography showed 18.8% pulmonary involvement. In pulmonary involvement, pulmonary nodule was the most frequently seen lesion (64.5%). The other findings of pulmonary involvement were 18.8% diffuse interstitial lung disease, 12.5% bronchiectasis, and 27.1% bullae-emphysema. The mean age of patients with pulmonary involvement was higher and as they smoked more. The pulmonary involvement was 5.2 times higher in patients with skin lesions because of arsenic. Diffusing capacity of lung for carbon monoxide was significantly lower in patients with pulmonary involvement.

**Conclusion:**

Besides lung cancer, chronic occupational inhalation of arsenic exposure may cause non-malignant pulmonary findings such as bronchiectasis, pulmonary nodules and diffuse interstitial lung disease. So, in order to detect pulmonary involvement in the early stages, workers who experience occupational arsenic exposure should be followed by diffusion test and high-resolution computed tomography.

Chronic arsenic exposure has an adverse effect on human health. Arsenic exposure can be by food, air and water. Following arsenic exposure, arsenic accumulates in the skin, hair and nail. In workplaces, industrial arsenic is used in semi-conductive production and pigments, mining activities and in the production of metals, pesticides, and fungicide products. Because of this, workers can be exposed to contaminated air (1). High levels of inorganic arsenic inhalation is a well-known cause of respiratory cancer (2).

Multi-systemic involvement in this condition has been well established, including mucocutaneous, cardiovascular, neurological and hepatic disorders, along with malignant changes (1,3). Malignant (lung cancer) and non-malignant [bronchiectasis, diffuse interstitial lung disease (ILD), chronic obstructive lung disease] effects of chronic arsenic exposure on pulmonary system have been shown (4,5,6,7,8). In the small number of case series describing non-malignant pulmonary involvement, high-resolution computed tomography (HRCT) showed an increased number of bronchiectasis and pulmonary artery dilatation cases (4,5). However, there are not a large number of case series in the literature referring to arsenic exposure pulmonary involvement evaluated by HRCT. That is why we evaluated arsenic-exposed workers even though there were no pulmonary symptoms to show the early stage lung findings.

## MATERIALS AND METHODS

### Patients

The study was approved by the local ethics committee. The findings of 256 workers who were admitted to our Hospital between 2011 and 2012, with a suspicion of occupational arsenic exposure, were extracted from prospectively collected hospital database records. In our study, patients were working in copper or lead melting, wooden repairing, or pesticide application, in which arsenic is either used or manufactured. We evaluated the arsenic levels in the hair and urine. Besides the demographic findings; clinical symptoms, working duration, skin and physical examination findings, blood and urine arsenic levels, HRCT, pulmonary function tests (PFTs), and arterial blood gas levels were recorded in the evaluation forms. None of the patients had pulmonary disease history like asthma, chronic obstructive pulmonary disease (COPD) and tuberculosis.

### Definition of as exposure

### Biochemical analysis

Morning urine samples were collected in 100 mL polypropylene tubes. Here, 5 mL 65% nitric acid was mixed with 2 mL hydrochloric acid and then completed to 100 mL with deionised water. Then, 10 µL of urine samples were acidified with 1900 µL of this acid mixture. After vortexing the 2000 µL acidified samples, they were sent for urine arsenic concentration determination using Inductively Coupled Plasma Mass Spectrometry (ICP-MS; 7700 Series; Agilent Technologies Inc., Tokyo, Japan).

Hair samples were collected from the nape of the neck and cut into approximately 0.5 cm pieces in length. The samples were thoroughly cleaned with 0.1% Triton-X solution and then washed several times with deionised water. Specimens were dried in an oven at 71 °C for 16 hours. Then, 0.1 g specimens were weighed and microwaved after the addition of 10 mL 65% nitric acid (CEM Mars Xpress microwave oven at 180 °C for 20 min) in high temperature-resistant Teflon tubes. After the burning process, we added 10 mL deionised water, mixed the sample and then transferred it to polypropylene tubes. The samples were analysed by the ICP-MS 7700 Series (Agilent Technologies Inc., Tokyo, Japan).

### Radiologic examination

### High-resolution computed tomography

All 256 of the arsenic-exposed workers had been evaluated with HRCT scans. The computerised tomography (CT) device was a GE HiSpeed which was a dual slice CT (GE Healthcare, Waukesha, U.S.A). The technique parameters were 1 mm thickness slices with 10 mm intervals, beginning from the apex to the base of the lung. Besides thin slices, bone algorithm reconstruction was used for high resolution. The images were evaluated on hard copy films as well as some of them on medical monitors which were in Picture Archiving and Communication Systems. Scanning parameters were 100 mAs, 130 kV and a 512x512 reconstruction matrix. CT examination was done in supine position with a deep inspiration. Hard copy films were obtained at a window of 1500 Hounsfield units (HU) and a level of -600 HU for parenchymal evaluation.

The lung parenchymal evaluations were done according to diffuse interstitial involvement, nodules, bronchiectasis, bullae and emphysema. Nine of the patients had diffuse pulmonary involvement, with all of them having micronodules. Although the micronodules were dominant in diffuse interstitial involvement, some patients also had reticular findings, in particular peripheral interlobular septal thickening. Besides diffuse involvement, some workers had nodules which were either individual or multiple in number, but not diffuse. This finding was categorised under pulmonary nodules. These nodules were less than 1 cm in diameter. In the pulmonary nodules category, 8 of the patients had a single nodule, while the rest had multiple nodules. These nodules were evaluated according to location, either upper, middle, or lower, and were assessed as unilateral or bilateral. Also, the location of pulmonary nodules was evaluated as parenchymal, subpleural or pleural. All of the bronchiectasis cases were in the mild category, where the bronchial diameter was 1.5-3 times the diameter of the accompanying artery (9).

### Pulmonary function tests

PFTs and carbon monoxide diffusion capacity (DLCO) were measured with a Zan 100 (nSpire Health Inc., Oberthulba, Germany) PFT device. PFTs were performed according to the American Thoracic Society Guidelines (10). PFTs were applied to 252 patients; four patients were not amenable to the test. The diffusion test was applied to 211 patients.

### Arterial blood gas

Arterial blood gas punctions were applied to the non-dominant hand radial artery without local anaesthesia. Oxygen tension in the arterial blood (PaO_2_), carbon dioxide tension in the arterial blood (PaCO_2_), percentage of haemoglobin saturated with oxygen in the arterial blood (SaO_2_) and the power of Hydrogen (pH) levels were measured with ABL 555 model device (Radiometer-Copenhagen, Denmark). Arterial blood gas measurements were taken for 198 patients.

### Statistical analysis

Cross tables were used for categorical variables. Numerical variables were presented as mean, median, standard deviation, minimum and maximum. For the comparison of categorical variables, either the Chi Square test or Monte Carlo Simulation was used. Bivariate analysis was performed with Fisher’s Exact test or the Mann Whitney U test. A logistic regression model was used in order to evaluate independent risk factors for pulmonary involvement. The independent effect of each variable on dependent variable was assessed with the multivariate logistic regression analysis backward conditional method. The model included age, cigarette smoking, and arsenic exposure evaluated by skin involvement and hair and urinary arsenic levels. The statistical significance level was p<0.05. All statistical analyses were performed using Statistical Package for the Social Sciences (SPSS) for Windows program, version 21 (SPSS Inc., Chicago, IL, USA).

## RESULTS

In this study, 256 male workers were included with an average age of 32.9±7.8 years. Exposure year average was 3.5±2.7. Hair and urine arsenic levels were 2.0±2.7 mg/L (n=238) and 43.2±26.2 (n=147) in order. Demographic profile, lung functions and laboratory results of the exposure group are shown in Table 1.

HRCT showed pulmonary involvement in 48 (18.8%) patients among the arsenic-exposed workers. Among the pulmonary involvement patients, 9 (18.8%) had diffuse interstitial disease, 6 (12.5%) had bronchiectasis, 31 (64.5%) had pulmonary nodules, and 13 (27.1%) had bulla-emphysema (Table 2). None of the pulmonary nodules were considered malignant because their diameter was less than 1 cm and all had regular contour.

The individuals with pulmonary involvement were older and smoked more compared to those with non-pulmonary involvement. Also, skin lesions were significantly higher in pulmonary involvement patients.

DLCO was significantly lower in pulmonary involvement patients.

Functional parameters and blood gas parameters were low in pulmonary involved patients, but the difference was not statistically significant (Table 1).

Among 252 workers, 18 (7.1%) had functional impairment. Among them, 15 (83.3%) were obstructive and 3 (16.7%) were restrictive. Among the 48 pulmonary involvement patients, 4 (8.3%) had pulmonary function impairment. All of them had the obstructive type. The ratio of patients with a DLCO abnormality to pulmonary involvement is 3 (10.7%).

In the model to determine the risk factors of pulmonary involvement showing age, skin lesions, and urine and hair arsenic levels, only skin lesions and age were found to be statistically significant risk factors (Table 3).

## DISCUSSION

Our wide case series of occupationally arsenic exposed workers is the only study showing either radiological (with HRCT) or functional (with DLCO) impairment in early stages. In our study, workers with skin lesions had a pulmonary involvement 5.2 times more often. DLCO was significantly lower in those patients who showed pulmonary involvement in their HRCT examinations. We showed the effects of arsenic exposure on pulmonary system in the early stages, either radiologically or functionally. The importance of our study comes from the widest series of HRCT-applied patients. HRCT was given to all exposed patients without looking at cutaneous and pulmonary symptoms. Our results therefore show that non-malignant pulmonary diseases (especially diffuse ILD, bronchiectasis, pulmonary nodule) are more in patients exposed to arsenic (respiratory) on an occupational basis.

Many studies showed that chronic arsenic exposure leads to respiratory effects. The first shown respiratory effect of arsenic was in West Bengal 1995. Coughing was reported in 57% of the 156 people living in villages affected by arsenic (11). In a study of 7.683 people, including both women and men, who were exposed to inorganic arsenic through drinking water, living in the West Bengal area of India, with the increasing arsenic concentration coughing, shortness of breath and auscultation findings also increased. Respiratory symptoms were more common in patients with skin lesions (12). Another study in West Bengal reported 107 patients with chronic arsenic exposure; 33 (30.8%) of them showed pulmonary involvement evidence. Pulmonary involvement patients were older, and had higher cutaneous scores and symptom scores (3).

The incidence of respiratory symptoms such as coughing, sputum, shortness of breath, and weakness were not significantly different between the groups of pulmonary involvement and non-involvement. This condition signifies the importance of HRCT to show the effects of arsenic on the lung even when there is no symptomatic lung disease. 

In the literature, studies have shown that arsenic exposure leads to a decrease in pulmonary function. Some studies in Sweden in the 1950s reported that arsenic exposure via inhalation caused non-malignant respiratory effects in copper casting workers. In one clinical study of 1.459 copper smelter workers, cited by Gerhardsson et al. (13), a syndrome characterised by chronic rhino- pharyngo- tracheobronchitis, lesions of the mucous membranes of the upper respiratory system, emphysema and decreased pulmonary function was described. In West Bengal, people were chronically exposed to arsenic via drinking water, so pulmonary involvement is evaluated objectively by looking at lung functions. The functional parameters [forced expired volume in first second (FEV_1_) and forced vital capacity (FVC), FEV_1_/FVC, peak expiratory flow rate] of pulmonary involvement patients (n=33) were significantly lower when compared to non-involvement patients (n=74). Pulmonary involvement patients were older and had high cutaneous and clinical scores. The arsenic concentration was high in consumed water. However, time of water drinking, and hair and nail arsenic levels were not different in either group (3).

In our study, there were no significant differences between pulmonary involvement and non-involvement according to functional parameters and blood gas parameters. However, DLCO was significantly lower in pulmonary involvement. Pulmonary involvement patients were older and smoked more. Skin lesions were significantly higher in pulmonary involvement patients. There was no significant difference between both groups according to hair and urine levels. These findings were similar to the findings in the literature. In the early stages of pulmonary involvement, because of the decrease in surface area of the alveolar capillary membrane where gas exchange occurs, a decrease in DLCO is seen (14). In the early stages of disease, DLCO decline may be the first and only finding. DLCO is the most sensitive test for evaluating respiratory functions in pulmonary involvement (15). A decline in DLCO can be evaluated as the earliest parameter in arsenic exposed pulmonary involvement. This result was not previously shown.

There are few studies where non-malignant effects of arsenic exposure on the pulmonary system were evaluated by HRCT (3,5,12). In a hospital-based study in Kolkata, West Bengal, non-malignant lung diseases were evaluated by lung radiography. The rate of non-malignant disease was 27.1% (29). After clinical evaluation, the patients were diagnosed as follows: 17 (58.62%) COPD cases, 3 (10%) bronchiectasis cases, and 9 (31.2%) ILD patients. However, only 4 were diagnosed with HRCT (3). In India, West Bengal, 56 patients with chronic coughing (41 exposure, 15 non-exposure) were studied, and the CT findings of either pulmonary artery dilatation or bronchiectasis scores were higher in chronic arsenic exposure patients (12). In another study, bronchiectasis was found 10 times more often in HRCT scans of patients with skin lesions because of arsenic exposure (15).

**Figure 1:**
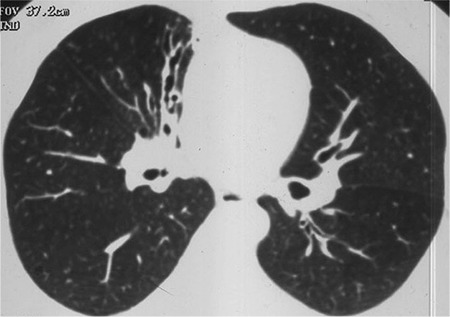
Bronchiectasis is seen at right paramediastinal area.

**Figure 2:**
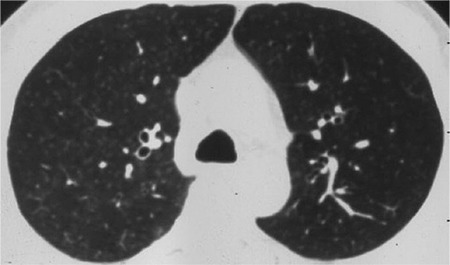
Bilateral diffuse interstitial micronodularity.

**Figure 3:**
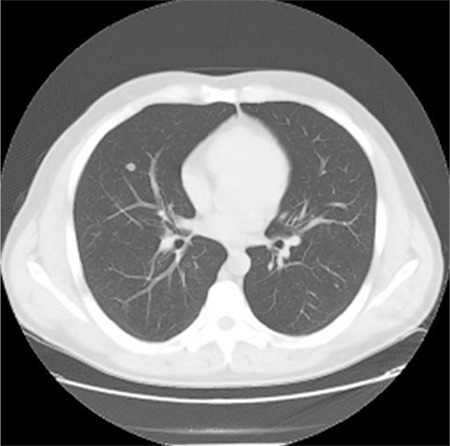
There is a parenchymal solitary pulmonary nodule in the middle lobe of the right lung.

In our study, pulmonary involvement was 48 (18.8%) in HRCT of arsenic-exposed patients. Pulmonary involvement was diffuse interstitial disease in 9 cases (18.8%), bronchiectasis in 6 cases (12.5%), pulmonary nodules in 31 cases (64.5%), and bulla-emphysema in 13 (27.1%) (Figure 1,2,3). We found pulmonary involvement 5.26 times more often in patients with skin lesions. In bronchiectasis, coughing was more prominent in acute stages, and if there was no exacerbation coughing may not be seen. In a West Bengal study, bronchiectasis cases were shown to be missed if there were no symptoms, which is why HRCT was suggested, without looking at the existence of skin lesions and pulmonary symptoms in arsenic-exposed patients. This allowed us to identify the pulmonary involvement, even in asymptomatic patients.

Arsenic is accepted as carcinogenic for the lung by the International Agency for Cancer Research (16). Different studies found that lung cancer incidence in arsenic-exposed people was 3.7% and 8.8% (3,17). The malignancy ratio of CT-detected pulmonary nodules was reported as 1.1-12% (18). In our study, the most frequent finding in HRCT was pulmonary nodules, giving support to the statement that arsenic-exposed patients should be followed more carefully.

The main limitation of our study was that 79% of pulmonary involvement workers were smokers. Smoking is one of the factors affecting DLCO. At least 24 hours before the DLCO test, patients should refrain from smoking; however, not all patients comply with this warning. As a result, smoking may contribute to the decline in DLCO in pulmonary involvement patients.

In conclusion, this study showed that occupational arsenic exposure increases the risk of non-malignant pulmonary diseases such as pulmonary nodules, ILD and bronchiectasis. HRCT and DLCO are very important in the early detection of pulmonary involvement, even when there are no pulmonary symptoms. We advise undertaking HRCT scans and DLCO to detect pulmonary involvement in the early stages when there is occupational arsenic exposure. At arsenic-exposed workplaces, any necessary safety precautions should be taken and the adverse effects of arsenic should be determined using appropriate screening programs.

## Figures and Tables

**Table 1:**
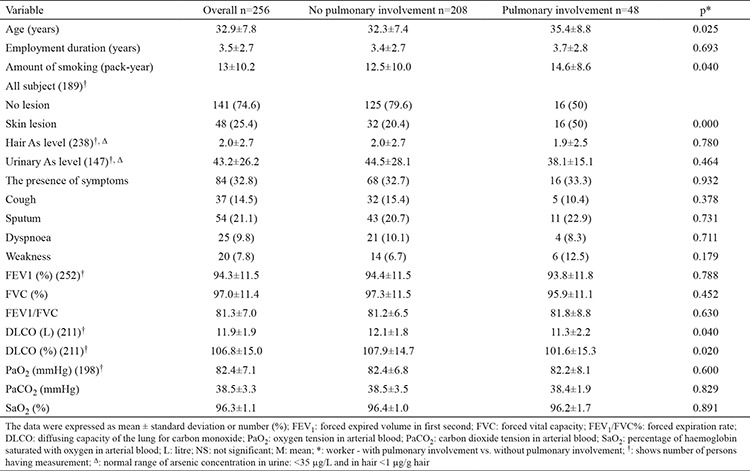
The comparison of workers with pulmonary involvement and those without pulmonary involvement in terms of demographic characteristics, pulmonary function tests and laboratory findings

**Table 2:**
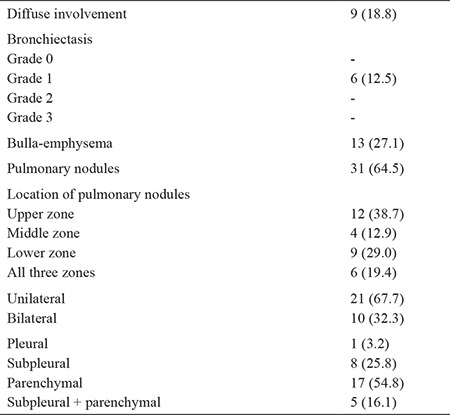
High-resolution computed tomography findings of pulmonary involvement (n=48) in arsenic exposed patients, All values are expressed as number (percentage)

**Table 3:**
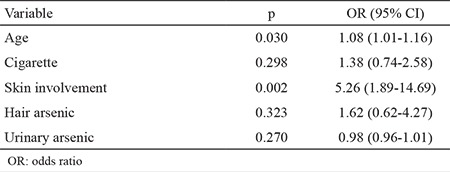
Risk factors for pulmonary involvement
